# The Melanin-Concentrating Hormone (MCH) System: A Tale of Two Peptides

**DOI:** 10.3389/fnins.2019.01280

**Published:** 2019-11-26

**Authors:** Giovanne B. Diniz, Jackson C. Bittencourt

**Affiliations:** ^1^Departamento de Anatomia, Instituto de Ciências Biomedicas, Universidade de São Paulo, São Paulo, Brazil; ^2^Department of Neurosurgery, Yale School of Medicine, New Haven, CT, United States; ^3^Nucleo de Neurociencias e Comportamento, Instituto de Psicologia, Universidade de São Paulo, São Paulo, Brazil

**Keywords:** NEI, MCHR1, MCHR2, lateral hypothalamus, neuropeptides, vertebrates

## Abstract

The melanin-concentrating hormone (MCH) system is a robust integrator of exogenous and endogenous information, modulating arousal and energy balance in mammals. Its predominant function in teleosts, however, is to concentrate melanin in the scales, contributing to the adaptive color change observed in several teleost species. These contrasting functions resulted from a gene duplication that occurred after the teleost divergence, which resulted in the generation of two MCH-coding genes in this clade, which acquired distinctive sequences, distribution, and functions, examined in detail here. We also describe the distribution of MCH immunoreactivity and gene expression in a large number of species, in an attempt to identify its core elements. While initially originated as a periventricular peptide, with an intimate relationship with the third ventricle, multiple events of lateral migration occurred during evolution, making the ventrolateral and dorsolateral hypothalamus the predominant sites of MCH in teleosts and mammals, respectively. Substantial differences between species can be identified, likely reflecting differences in habitat and behavior. This observation aligns well with the idea that MCH is a major integrator of internal and external information, ensuring an appropriate response to ensure the organism’s homeostasis. New studies on the MCH system in species that have not yet been investigated will help us understand more precisely how these habitat changes are connected to the hypothalamic neurochemical circuits, paving the way to new intervention strategies that may be used with pharmacological purposes.

## Introduction

### The Melanin-Concentrating Hormone System

The melanin-concentrating hormone (MCH) system is a robust integrator of exogenous and endogenous information, modulating arousal, promoting motivated behaviors, and controlling energy balance (Diniz and Bittencourt, 2017; Chee et al., 2019), contributing to the appropriate sleep architecture (Ferreira et al., 2017a; Gao, 2018), and tethering energy status and reproductive physiology (Naufahu et al., 2013). While certain aspects of this system have been explored in length, such as its neuroanatomical aspects (Bittencourt and Diniz, 2018), several others are still open to investigation, including its role in parental behavior (Benedetto et al., 2014; Alachkar et al., 2016; Alhassen et al., 2019), or the mechanisms through which MCH is used to convey information within the central nervous system (CNS) (Noble et al., 2018). Although MCH is strongly linked to the roles above, its discovery, and hence its name is linked to an additional function performed in Teleosts: the control of skin color through the modulation of chromatophore activity (Kawauchi et al., 1983). MCH is synthesized by neurons in the Teleost hypothalamus and released in the bloodstream through the neurohypophysis (NH), conferring MCH the status of neurohormone. Upon reaching the melanophores in the scales, MCH promotes pallor, necessary for adaptive color change. Although only isolated in 1983, its existence was predicted almost 50 years prior, by Hogben and Slome (1931).

**FIGURE 1:**
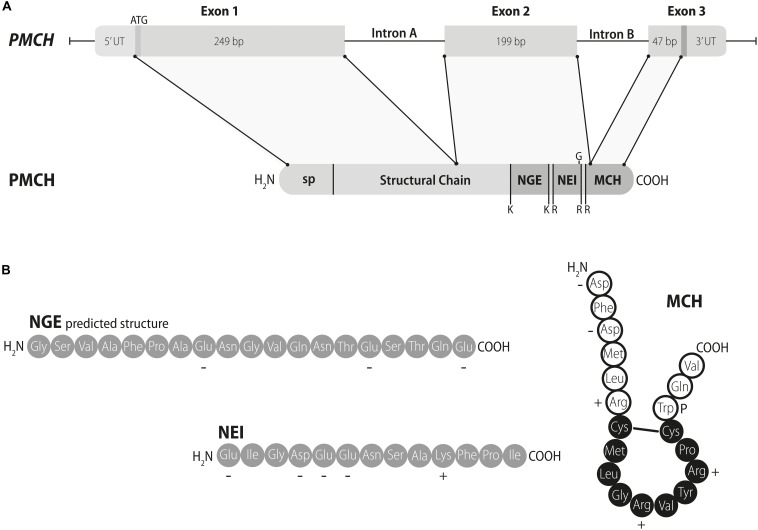
The mammalian *PMCH* gene, PMCH protein, and the peptides of the MCH family. **(A)** The mammalian PMCH gene is composed of three exons and two introns. Both introns and exons have variable lengths, with the sizes depicted in this diagram corresponding to human *PMCH*. The transcribed product of *PMCH* is formed by five components: a signal peptide (sp), encoded in Exon 1; a structural chain, formed by both Exons 1 and 2; two MCH gene-related peptides (MGRPs), neuropeptide G-E (NGE) and neuropeptide E-I (NEI), both encoded in Exon 2; and a mature melanin-concentrating hormone (MCH), with 3 N-terminal residues found in Exon 2, a residue formed by Exon 2 and Exon 3 combining to generate its codon, and 15 C-terminal residues found in Exon 3. Both MCH and NEI can be generated through dibasic proteolytic sites, indicated in the diagram by RR and KR, and NGE may be cleaved at a single basic site K. Neuropeptide E-I contains an amidation site in its C-terminal portion, indicated by G. **(B)** Structure of the peptides generated from *PMCH*. Both NGE and NEI are linear peptides characterized by a predominance of negatively charged residues. The precise structure of NGE is unknown, given that this peptide has never been isolated and characterized. Mature MCH, on the other hand, is a cyclic peptide, due to a cysteine bridge formed between residues 7 and 16. The ring structure formed between the two cysteine residues, indicated by black circles, is essential for the binding activity of MCH to its receptors. Residue 17 plays an important role in potentiating MCH binding (indicated by the letter P). Adapted with permission from Bittencourt and Diniz (2018).

The identification of MCH in the chum salmon pituitary was the gateway for a plethora of discoveries regarding this system. Just six years after its description in Salmoniforms, Nahon et al. (1989) identified the mammalian *Pmch* gene, as well as other predicted peptides that originate from the *Pmch*-encoded precursor, PMCH: neuropeptide E-I (NEI) and neuropeptide G-E (NGE), following the nomenclature scheme of Tatemoto and Mutt (1981) (Figure 1). In that same year, Vaughan et al. (1989) isolated and sequenced the mammalian MCH peptide (Figure 1). In parallel, different peptides were identified originating from the teleost *Pmch* gene, such as neuropeptide E-V (Minth et al., 1989). Three years later, Bittencourt et al. (1992) published the first complete mapping of *Pmch* expression and MCH and NEI immunoreactivity (IR) in the rat brain.

While a wide range of vertebrates has been investigated in terms of *Pmch* expression and MCH synthesis, the precise origin of *Pmch* in vertebrates is unknown. As we will see in detail in the next section, lampreys are the earliest-diverging animals with evidence of MCH existence. Nahon et al. (1989) proposed that PMCH and the prepropeptide-A of the sea slug *Aplysia californica* are distantly related, given the 24% of sequence identity shared between the two prepropeptides and the matching sets of cleavage sites. This hypothesis, however, failed to gain traction in the literature, making the origin of *Pmch* in vertebrates an important open question that will require experimental investigation to be answered. In the lack of new data, the most parsimonious hypothesis is that the founder gene of *Pmch* originated in phylostratum 11, as determined by the phylostratigraphy method of Domazet-Lošo and Tautz (2010) and Domazet-Lošo et al. (2007).

The next breakthrough in the field came at the turn of the century, when reverse pharmacology studies identified GPR24/SLC-1 as the selective receptor for MCH, now known as MCHR1 (Bächner et al., 1999; Chambers et al., 1999; Lembo et al., 1999; Saito et al., 1999; Shimomura et al., 1999). Homology searches in genomic databases then revealed a second MCH receptor in 2001, now known as MCHR2 (An et al., 2001; Hill et al., 2001; Mori et al., 2001; Rodriguez et al., 2001; Sailer et al., 2001; Wang et al., 2001). Initially identified as the somatostatin-like coupled receptor 1, MCHR1 shares over 40% identity with somatostatin receptors (SSTRs) in the transmembrane domains (Kolakowski et al., 1996; Lakaye et al., 1998). Despite these similarities, somatostatin does not bind to MCHR1, with MCH acting as its only specific ligand. The level of similarity between MCHR1 and SSTRs is comparable to the identity between the paralogs MCHR1 and MCHR2, which share 44% identity in the transmembrane domain (An et al., 2001). As is the case with *Pmch*, the precise origin of MCHR-coding genes in the vertebrate lineage is unknown. Yun et al. (2015) suggested that putative ortholog genes of *Mchr* exist in the ascidian *Ciona intestinalis* and in the lancelet *Branchiostoma floridae*. Furthermore, in the same work, *B. floridae* and vertebrate *Mchr* paralogs were nested with the protostome neuropeptide receptor family 24 (*Npr-24*) of *Caenorhabditis elegans*, suggesting a possible distant relationship between MCH receptors and *Npr-24*. This falls in line with the idea that many neuropeptidergic families have roots in invertebrate species (Elphick et al., 2018).

In addition to the canonical MCH system, there are non-canonical transcripts that originate from the *Pmch*/*PMCH* genes. Toumaniantz et al. (1996) discovered an alternative-splicing product originating from those genes, the MCH-gene-overprinted-polypeptide (MGOP). In the antisense strand of the *Pmch/PMCH* genes, Borsu et al. (2000) identified the antisense-RNA-overlapping-MCH (AROM), a complex gene that originated coding and non-coding transcripts that may modulate gene expression (Moldovan et al., 2012). Finally, exclusively in the hominid lineage, two chimeric genes originated from *PMCH, PMCH-Linked* 1, and 2 (*PMCHL1/PMCHL2*), with putative transcription modulation activity (Courseaux and Nahon, 2001).

As just a glance reveals, MCH is part of a complex system, involved in numerous functions and with multiple canonical and non-canonical elements. This complexity stems from a rich evolutionary history, as significant genomic events influenced the *Pmch* gene and its ancillary elements. The MCH system, therefore, provides us with a window to look at those evolutionary events and how they shaped the vertebrate hypothalamus and its circuits. In this review, the canonical MCH peptidergic system will be reviewed, including the orthologs and paralogs of the *Pmch* gene, and the distribution of the *Pmch*-coded peptides within the nervous system. Due to the abundance of data, we will not include the MCH receptors and the non-canonical elements, except when they help us understand the peptidergic family. A brief description of the phylogenetic relationship between major clades is included in each section to help readers contextualize the information.

## The Mch System in Early Chordates

### Chordata > Petromyzontiformes

**FIGURE 2:**
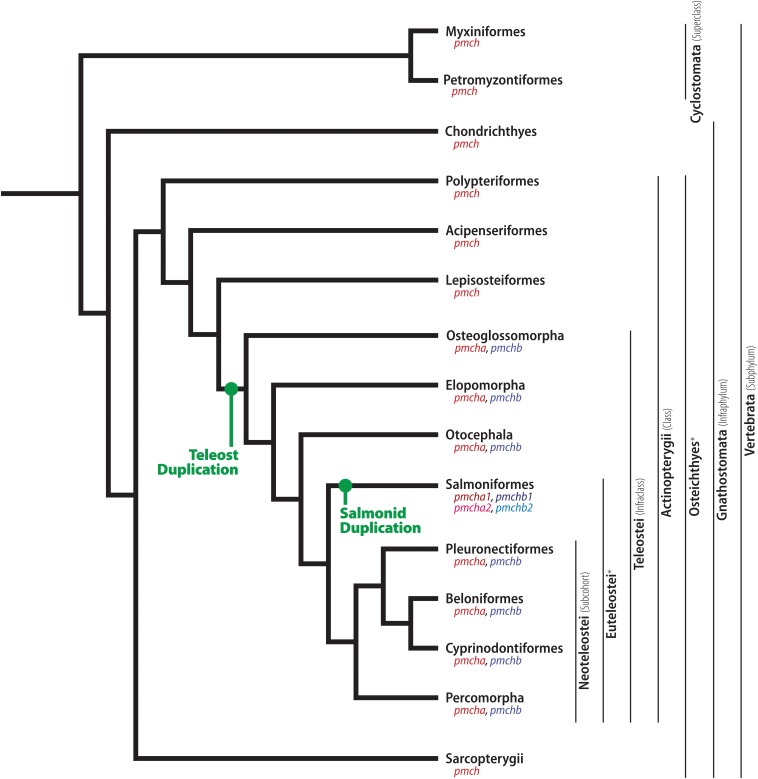
Phylogenetic relationship between early-diverging Chordates and Actinopterygians. Illustrative cladogram showing the main phylogenetic relationship between clades with available information about MCH. Several clades have been omitted for clarity, and the cladogram is not meant to represent the absolute order of relationship between these groups, but rather the most probable organization based on current molecular data and information about the MCH system. The relative position of two *pmch* duplication events are indicated in green, and the resulting genes are indicated under each clade name. The ubiquitous vertebrate *pmch* and its homologs are indicated in red, while the teleost-specific *pmchb* is indicated in blue. The duplicated copies originated in the salmonid lineage are indicated in different shades of red and blue. To facilitate the reading of this review, relevant clades are indicated alongside their rank on the **right** side of the cladogram, encompassing the clades to the **left** of each black line. ^∗^ Unranked clades.

Lampreys (order Petromyzontiformes) and hagfishes (order Myxiniformes) were the first vertebrates to diverge (Figure 2), with molecular estimates ranging from 797 to 489 MYA (Kumar and Hedges, 1998; Blair and Hedges, 2005; Kuraku and Kuratani, 2006; Kumar A. et al., 2017; Miyashita et al., 2019), while fossil data situates this divergence predating the Devonian, at least 360 MYA (Gess et al., 2006). Together, these two groups form superclass Cyclostomata, clade characterized by the lack of a jaw and internal branchial arches. Due to the very early divergence of Cyclostomata, species belonging to this clade are often considered models for the common vertebrate ancestor. Four species of lamprey have been used to study MCH: the European river lamprey (*Lampetra fluviatilis*), sea lamprey (*Petromyzon marinus*), brook lamprey (*Lampetra planeri*), and pouched lamprey (*Geotria australis*). Morphological data has been obtained in all species using a salmon MCH-directed antibody (Al-Yousuf and Mizuno, 1991; Bird et al., 2001). Little is known about MCH-related genes in Petromyzontids, despite the availability of the whole genome of *P. marinus* (Smith et al., 2013). Two genes are annotated as *Mchr1* (ENSPMAG00000005548.1) and *Mchr2* (ENSPMAG00000001735.1), but there is no gene annotated as *Pmch*. It should be noted, however, that scaffold GL478617 contains a sequence that codes for a peptide that has 13 identical residues when compared to Gnathostome *Pmch*, including a fully conserved cysteine ring, but experimental confirmation is necessary.

**FIGURE 3:**
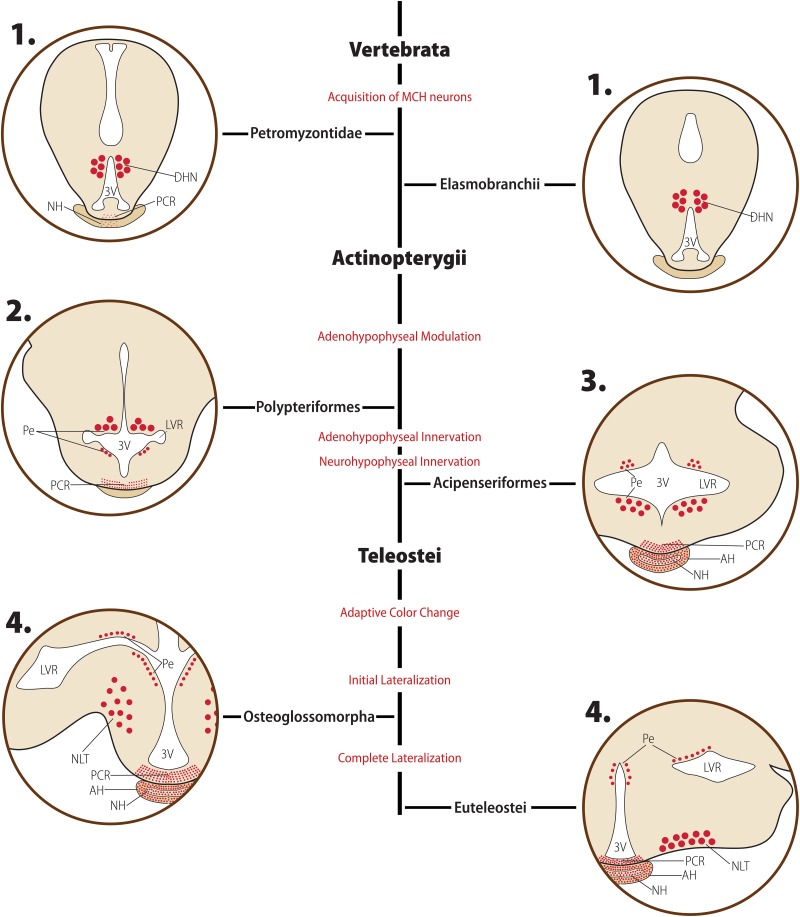
Diagrammatic representation of the main morphological features of the MCH distribution in early-diverged Chordates and Actinopterygians. Major clades are indicated in bold font, while clade-wide events are represented in red. The diagrams for each clade are not meant to represent a single species or a single hypothalamic level, but rather a visual summary of what has been described for animals belonging to that clade. Red circles represent MCH neurons: small circles represent neurons with small somas, while large circles represent neurons with enlarged somas, typically associated with a neurosecretory profile. Fiber plexuses are represented by red dots. **1.** In Petromyzontids and Elasmobranchs, the majority of MCH neurons is found in the ependymal and subependymal layers (Pe) of the third ventricle (3V), within the dorsomedial hypothalamic nucleus (DHN). While there is no indication of hypophyseal innervation in Elasmobranchs, there are mixed reports about the presence of MCH in the proximal neurosecretory contact region (PCR) and neurohypophysis (NH) of Petromyzontids. **2.** After the Actinopterygii divergence, two major changes occurred in the MCH system. First, the periventricular neurons were divided in two groups by the emerging lateral recess, separating them into dorsal and ventral groups. Second, a clear innervation of the PCR develops, indicating the acquisition of a modulatory role over the adenohypophysis by MCH neurons at this stage. **3.** Shortly after the development of PCR innervation, MCH fibers started innervating the adenohypophysis (AH) and NH directly, creating the anatomical substrate necessary for a neurosecretory role. **4.** Around the time of the Teleost split, the final large-scale changes in the MCH system occurred. A lateral migration process started, with large, neurosecretory MCH neurons moving toward the *nucleus lateralis tuberis* (NLT) and densely projecting to the hypophysis from this ventrolateral position. This change occurred contemporaneously to the acquisition of a role in adaptive color change by MCH. Based on data from [opetwcite]B16,B51[clotwcite]Baker and Bird (2002); Duarte et al. (2001) and Amano et al. (2003).

In Petromyzontids, MCH-immunoreactive cells are predominantly restricted to a single hypothalamic *locus*, the dorsomedial hypothalamic nucleus (DHN) of the posterior hypothalamus, occupying parts of the ependyma and subependyma, with only a few neurons found scattered toward the lateral hypothalamus (LH) (Al-Yousuf and Mizuno, 1991; Bird et al., 2001). The position of MCH cells in the hypothalamus is illustrated in Figure 3. The DHN is found bordering the third ventricle (3V) and is part of the paraventricular organ (PVO), an ubiquitous non-mammalian structure that contains a myriad of neuroactive substances (Nozaki et al., 1983; Brodin et al., 1990; Tobet et al., 1995), plays a role in hypothalamic integration (Vigh-Teichmann and Vigh, 1989; Meurling and Rodríguez, 1990), has no blood-brain barrier, and is highly vascularized (Röhlich and Vigh, 1967). Exclusively in sexually maturing *L. petromizon*, a weakly-labeled group of neurons is found in the anterior basal telencephalon. Neurons immunoreactive to MCH in the petromyzontid DHN are frequently bipolar, with one axon projecting into the 3V *lumen*, and the other axon extending laterally toward the LH.

In addition to local lateral projections, three major innervation pathways are observed: anterior, toward the olfactory lobes; dorsal, toward the habenular nucleus; and posterior, toward the spinal cord (Bird et al., 2001). In *L. fluviatilis* and *L. planeri*, an additional innervation pathway is observed: ventral, toward the hypophysis. In lamprey, the hypophysis is continuous with the hypothalamic floor, as there is no portal system or *infundibulum*. In *L. fluviatilis*, immunoreactive fibers are observed in the proximal neurosecretory contact region (PCR) – the Petromyzontid homolog of the tetrapod median eminence (ME) – and in the proximal NH. Some immunolabeled axon terminals are also found close to the basement membrane that separates the NH and the *pars distalis* of the adenohypophysis (AH) (Al-Yousuf and Mizuno, 1991). It should be mentioned, however, that Baker and Rance (1983) did not find evidence of MCH activity in a bioassay using *L. fluviatilis* hypophyses.

Based on these data, Bird et al. (2001) make a series of morphofunctional correlates that, as it will be described later in this work, are extremely relevant for the understanding of MCH in mammals. These authors suggest that: (1) the 3V-contacting axon of MCH neurons, and their position near the ependyma and subependyma, could allow those neurons to sense biomarkers in the cerebrospinal fluid (CSF), or release MCH directly in the *lumen* to act on distant sites; (2) The ample distribution of laterally-projecting axons allows MCH neurons to exert widespread modulation within the CNS; (3) The moderate presence of MCH-ir terminals in the NH could be the substrate through which MCH modulates the release of other neuropeptides in a paracrine fashion, or influences the secretory action of the AH; (4) The NH MCH fibers observed in *L. petromizon* could originate from the telencephalic group of cells observed only in sexually maturing individuals of that species, and those neurons could play a role in physiological adaptation toward reproduction.

### Chordata > Gnathostomata > Chondrichthyes

Cartilaginous fish, members of the Chondrichthyes class, are the earliest diverging class of living jawed vertebrates (Gnathostomata), splitting from bony vertebrates (Osteichthyes) between 475 and 450 MYA (Venkatesh et al., 2014; Kumar S. et al., 2017; Figure 2). Extant animals are divided into two subclades, Holocephali (chimeras) and Elasmobranchii (sharks and rays), which split about 421 MYA (Renz et al., 2013).

Chondrichthyans have a single *pmch* gene composed of three exons, encoding a Pmch precursor that is 172 aa-long in the Holocephalan Australian ghost shark (*Callorhinchus milii – RefSeq: XP_007893646.1*) or 165 aa-long in the Elasmobranch scalloped hammerhead (*Sphyrna lewini – BAM63324.1*). At the N-terminus of this precursors sits a signal peptide that varies in length according to the species, and at the C-terminus is a mature 19 aa-long MCH that can be released by proteolytic activity through a dibasic Arg-Arg site (Mizusawa et al., 2012). In *C. milii*, it is possible that a second 14 aa-long peptide (Neuropeptide G-T) is cleaved from another dibasic pair upstream from the MCH-originating pair, but in *S. lewini* this peptide is 33 aa in length (Neuropeptide T-V). Since there is significant variation in the other peptides that may be produced from the *pmch* gene in addition to MCH, these peptides will all be grouped under an umbrella term: MCH gene-related peptides (MGRPs), a term borrowed from Gröneveld et al. (1993). The Chondrichthyan *pmch* gene is a perfect blueprint for the mammalian *PMCH*. Both human and *S. lewini* precursors have the same length (165 aa), a signal peptide in its N-terminus (which varies in length depending on the species), and a 19 aa-long mature MCH in the C-terminus that can be cleaved from an Arg-Arg pair. There is a single substitution between mammalian MCH and Chondrichthyes MCH (Val^19^ in most mammals, Asn^19^ in *S. lewini*, Ile^19^ in *C. milii*), but the ring structure between Cys^7^-Cys^16^ is wholly preserved. The conservation of the ring sequence is likely linked to its importance for MCH binding to its receptors (Macdonald et al., 2000).

Regarding the distribution of immunoreactivity in Chondrichthyes, labeled cells are found in the dorsal wall of the posterior hypothalamus, and fibers were found exclusively inside the hypothalamus (Mizusawa et al., 2012; Figure 3). This distribution of MCH-synthesizing neurons is similar to what has been described for Cyclostomes, further reinforcing the dorsomedial posterior hypothalamus as the original *locus* of MCH synthesis in the chordate brain. No fibers are found in the hypophysis, and it is unclear if the hypothalamic-restricted distribution of fibers is a feature of the species or a methodological artifact.

## The Mch System in the Ray-Finned Fish Lineage

### Gnathostomata > Osteichthyes > Actinopterygii

The superclass Osteichthyes contains all vertebrates with bony skeletons, splitting from Chondrichthyans at around 450 MYA (Venkatesh et al., 2014). Osteichtyans split early into two major clades, Actinopterygii (the ray-finned fishes) and Sarcopterygii (lobe-finned fishes), at around 435 MYA (Figure 2; Kumar S. et al., 2017). Actinopterygians then diverged into more than 25,000 known extant species, making it the most specious vertebrate clade, partially thanks to the Teleost radiation that occurred at the Cretaceous-Paleogene transition (Friedman, 2010; Sibert and Norris, 2015). Actinopterygians split into five clades: Polypteriformes, Acipenseriformes, Lepisosteiformes, Amiiformes, and Teleostei (Figure 2). There is uncertainty in the literature regarding the exact relationship between the clades of Actinopterygii (Kumar S. et al., 2017), but for this work it is only relevant that Polypteriformes, Acipenseriformes, Lepisosteiformes, and Amiiformes are all considered ancient with respect to Teleostei. Due to the importance of the latter to the understanding of MCH, those animals will be described in a separate section.

Regarding the makeup of *pmch* genes, there are no significant changes in non-Teleost Actinopterygii. The genes with available data are comprised of three introns, giving origin to 167–170 aa-long precursors (*Lepisosteus oculatus* – XP_015207385.1, *Erpetoichthys calabaricus* – XP_028672358.1). Mature MCH is 19 aa in length, with some divergence observed in the last residue (Val^19^ in *L. oculatus*, Ile^19^ in *E. calabaricus*), with a 13 aa-long MGRP possibly cleaved at an upstream Arg-Arg pair. Contrasting to the relatively unchanged genomic structure, the Actinopterygii divergence was a period of change in terms of the neuroanatomy of the MCH system. In the brain of the Polypteriform *E. calabaricus*, MCH IR is remarkably similar to what has been described for Petromyzontids. All immunoreactivity resides in the periependymal area, over the dorsal surface of the lateral ventricular recesses (LVR) and in the lateral wall of the 3V (Figure 3). These neurons are found within the PVO, close to the blood capillaries and contacting the ventricular cavity. Abundant fibers are found in the PCR, but not in the hypophysis (Baker and Bird, 2002). The distribution of immunoreactivity in the Acipenseriform starry sturgeon (*Acipenser stellatus*) is similar to that of *E. calabaricus*, with one striking difference: instead of stopping at the PCR, fibers continued toward the NH (Baker and Bird, 2002; Figure 3).

In the brain of the Lepisosteriform longnose gar (*Lepisosteus osseus)*, a significant change occurred. Instead of being concentrated in a single area, MCH neurons are found in two separate groups: the dorsomedial ventricular group, similar to what has been described previously, and a new group of neurons within the *nucleus lateralis tuberis* (NLT). Neurons in the ventricular group follow the same pattern as described for MCH neurons so far: predominantly bipolar, in association with the PVO, with one axon contacting the ventricular cavity and another branching in the lateral hypothalamus and other areas of the CNS. Fibers from the NLT, on the other hand, are found coursing through the basal hypothalamus toward the pituitary stalk, forming a plexus around blood capillaries of the PCR and NH (Baker and Bird, 2002).

The morphological aspects of the MCH system in the three aforementioned species are in accordance to some models of Actinopterygii divergence. Polypteriforms were likely the first clade to split, at around 407 MYA (Kumar S. et al., 2017), and the distribution of MCH-immunoreactivity in these animals closely resemble that of Petromyzontids and Elasmobranchii.

The next clade to split was likely Acipenseriformes, since *A. stellatus* has a very similar distribution of cellular bodies but differs from Polypteriforms by having a dense direct innervation of the NH. Since the lamprey *L. planari* also has a direct innervation of the NH, it is possible that this feature appeared independently in Petromyzontids and the common ancestor between Acipenseriforms and Lepisosteriforms. Alternatively, the innervation of the NH by MCH fibers may have first appeared in Petromyzontids as a plastic feature, becoming then fixed by the time of Acipenseriform divergence. An evolutive advantage of a direct NH innervation (interpreted as MCH release directly in the bloodstream) may have paved the way for the split between periventricular/dorsomedial and tuberal lateral groups to be positively selected, with NLT neurons becoming a magnocellular group that preferentially innervates the NH, which was then itself followed by the acquisition of adaptive color change.

### Actinopterygii > Teleostei

The infraclass Teleostei split from other Actinopterygii at around 320 MYA (Kumar S. et al., 2017). Teleosts can be divided into four major clades: Osteoglossomorpha, Elopomorpha, Otocephala, and Euteleostei (Figure 2). Most teleost species are part of Euteleostei, with extant members of the other three clades including bonytongues (Osteoglossomorpha), eels (Elopomorpha), and catfishes (Otocephala). Although there is some controversy regarding the exact phylogenetic relationship between Teleost clades, molecular and morphological data suggest that Osteoglossomorpha may have been the first clade to split, at 285 MYA, followed by Elopomorpha (265 MYA), and finally Otocephala and Euteleostei (230 MYA) (Kumar S. et al., 2017). Since several developments occurred in the MCH system shortly after the Teleostei split, and Euteleosts have been extensively used to probe the MCH system, they will be examined in the following section, with this section focusing on non-Euteleost Teleosts.

#### Structural and Evolutionary Aspects

Considerable changes in terms of genomic makeup marked the divergence of Teleosts. This period was characterized by accelerated genome changes, including higher rates of gene-linkage disruption and chromosomal rearrangements (Ravi and Venkatesh, 2008), a large number of retroposition events (Fu et al., 2010), and a teleost-specific whole-genome duplication event (Jaillon et al., 2004; Meyer and Van de Peer, 2005). A second copy of *pmch* was generated during this period, likely through retroposition, given that the newly generated copy is intronless, a hallmark of retrocopies (Fu et al., 2010; Grzybowska, 2012; Casola and Betrán, 2017), and does not follow the expected chromosomal location for a copy originated from a whole-genome duplication (Kasahara et al., 2007). This duplication, when compounded with the historical order of MCH discoveries, creates substantial clutter in the nomenclature of *pmch* orthologs and paralogs. Therefore, we will use in this review a nomenclature that adheres to the prescribed gene nomenclature guidelines for the various species examined, at the expense of not using some of the original nomenclature used in the literature. A table of normalized terms is provided (Table 1). Henceforth, the gene most commonly identified as “pmch2” will be designated as *pmcha*, and “pmch1” will be designated as *pmchb*. This nomenclature revision has the added benefit of conciliating the nomenclature for the mature MCH produced by teleost paralogs (MCH_A_ and MCH_B_) with the standardized nomenclature for MCH receptors (MCH_1_ and MCH_2_) as defined by the Nomenclature Committee of the Union of Basic and Clinical Pharmacology (NC-IUPHAR) (Alexander et al., 2017).

**TABLE 1:** Standardized nomenclature of *pmch* genes across vertebrate species.

**Gene abbreviation**	**Protein abbreviation**	**Diff. criteria**
**Petromyzontid**		
*Pmch*	Pmch	–
**Elasmobranchii**		
*pmch*	Pmch	–
**Actinopterygii (non-teleost)**		
*Pmch*	Pmch	–
**Teleost (non-salmonids)**		
*Pmcha*	Pmcha	Three exons
*pmchb*	Pmchb	One exon
**Salmonids**		
*pmcha1*	Pmcha1	Three exons, MCH (Ser^19^)
*pmcha2*	Pmcha2	Three exons
*pmchb1*	Pmchb1	One exon, NEV (Gly^3^)
*pmchb2*	Pmchb2	One exon
**Dipnoi**		
*Pmch*	Pmch	–
**Lissamphibian**		
*Pmch*	Pmch	–
**Sauropsid (non-aves)**		
*Pmch*	Pmch	–
**Aves**		
*PMCH*	PMCH	–
**Mammalian (non-primate)**		
*Pmch*	PMCH	–
**Primate**		
*PMCH*	PMCH	–

**The gene nomenclature guidelines used to compose this table are: [opetwcite]B122,B72[clotwcite]Sprague et al. (2006); Karimi et al. (2017), [opetwcite]B83,B33[clotwcite]Kusumi et al. (2011); Burt et al. (2009), Blake et al. (2016) and Braschi et al. (2018).**

The *pmcha* gene has a similar structure to both Elasmobranch and Mammalian *pmch/Pmch*, being comprised of three exons, having a 3’ splice site of intron 2 in the same position, and displaying similar synteny. The Pmcha prepropeptide ranges from 147 to 157 aa in length (*Electrophorus electricus* – XP_026877406.1, *Paramormyrops kingsleyae* – XP_023672675.1), and through a dibasic cleavage site originates a mature MCH_A_ that is 19 aa-long and 84.2% identical and 89.5% similar to mammalian MCH. Three substitutions are observed in the mature MCH_A_, Ile^2^ replaces Phe^2^, Val^9^ replaces Leu^9^, and Ala^19^ replaces Asn^19^/Ile^19^ in Elasmobranchii or Val^19^ in Mammals. An additional aa substitution may have occurred in the common carp, *Cyprinus carpio* (Ile^4^ replacing Met^4^ – XP_018948604.1, XP_018948612.1). These changes are all conservative, and only the Val^9^ substitution has occurred in the bioactive zone of MCH_A_. The *pmchb* gene, on the other hand, is intronless and codes a Pmchb precursor that is 124 aa-long in all described species (*C. carpio* – XP_018967332.1). There is a remarkably low similarity between *pmcha* and *pmchb*. At the C-terminus of Pmchb is a mature MCH_B_ that is 17 aa-long (two residues shorter in the N-terminus), has two substitutions on the N-terminal stretch before the ring structure (Thr^2^ replaces Met^4^ and Met^3^ replaces Leu^5^), the same substitution as MCH_A_ inside the ring, and one non-conservative substitution in the C-terminal sequence outside the ring (Glu^16^ replaces Gln^18^).

Two aspects of the formation of MCH_A_ and MCH_B_ in the Teleost ancestor are worth noting. The first is the remarkable capability of neuromodulators to change shortly after being duplicated. Given the uniform distribution of *pmchb* in early Teleosts, it is clear that this newly generated copy underwent its substitutions before there were any significant splits in the Teleost lineage. The second remarkable aspect is how genomic duplications affect phylogenetic constraints. As the only source of MCH in non-Teleosts, the *pmch/Pmch* gene remained remarkably conserved from Elasmobranchs to Mammals. In the Teleost lineage, however, *pmcha* became a very dynamic neuropeptide, while *pmchb* became the most conserved paralog, even though its sequence differs significantly from non-Teleost *pmch/Pmch*, probably due to the acquisition of an adaptive color change role for MCH, which then acted to impose a phylogenetic constraint over *pmchb*.

It should be mentioned that attempts to clone the transcripts of *pmch* genes in some Otocephala species have not always resulted in the identification of two paralogs. In the goldfish *Carassius auratus*, two paralogs encoding highly similar *pmch* were found (Cerdá-Reverter et al., 2006), and a single paralog was found in *Schizothorax prenati* (Wang et al., 2016) and *C. carpio* (Xu et al., 2019). This apparent contradiction between the genomic databases and the attempts to clone *pmch* transcripts can be explained by a very low expression of *pmcha*, coupled to a high dissimilarity between *pmcha* and *pmchb*. Since most probes have been designed based on the well-known salmon sequence (of *pmchb*), it is easy to imagine that most probes would fail to identify *pmcha* transcripts. Furthermore, as we will see in the next section, the use of different antibodies to map MCH in goldfish is supportive of the existence of two structurally dissimilar MCH peptides.

#### Anatomy

In the Osteoglossomorphs goldeye (*Hiodon alosoides*) and freshwater butterflyfish (*Pantodon buchholzi*), the bulk of MCH neurons is found in the basal hypothalamus, but instead of forming a neuronal sheet in the NLT area, neurons are found in the mid-hypothalamic region (Figure 3). Despite this difference, these neurons project to the NH, similar to the NLT group of other Actinopterygians. A second group of small neurons is found in the periventricular area of the LVR, clustered around the PVO. As the other periventricular groups described here, these neurons project to the ependyma and the ventricular cavity (Baker and Bird, 2002). In the Elopomorph European eel (*Anguilla anguilla*), 80% of MCH neurons are large and located in the NLT at the ventrolateral hypothalamus, below the LVR, with dense projections to the NH. This is the first significant shift of cells to the ventral hypothalamus from the periventricular area, a transitional stage that will be repeated in several other species. A small group of neurons still resides at the dorsal surface of the LVR, but there is no apparent contact with the PVO (Baker and Bird, 2002). In Otocephala, the dominant group of MCH neurons is located in the NLT, with dense projections to the hypophysis, in addition to projections to the thalamus, pretectal region, preoptic area, and telencephalon. A second, small group of neurons is observed close to the LVR, near the junction between the LVR and 3V. The axons of those neurons course toward the vicinity of the PVO, but no direct contact with the ventricular cavity is observed (Bird et al., 1989; Baker and Bird, 2002). These descriptions were made using a salmon MCH-directed antibody; therefore, those descriptions are likely more relevant to MCH_B_ than to MCH_A_ in those species. Fortunately, some works provide some insight into the differences between MCH_A_ and MCH_B_ in terms of distribution.

In zebrafish (*Danio rerio)*, *pmchb* mRNA expression is found in the lateral and posterior NLT, in a group dorsal to the LVR, and the caudal zone of the periventricular hypothalamus. The distribution of *pmcha*, on the other hand, is more restricted, with *pmcha*-expressing neurons found exclusively in the anterior NLT. There is no overlap in the expression of the two *pmch* paralogs. Immunoreactivity to MCH_A_ and MCH_B_ was determined to follow a similar pattern, as revealed by the use of salmon MCH- and mammalian MCH-directed antibodies. An extensive network of MCH_A_-ir fibers was found, including immunoreactivity in the dorsal nucleus of the ventral telencephalic area, the thalamus, the habenula, the periventricular nucleus of the posterior tuberculum, the posterior tuberal nucleus, and the *torus lateralis*. In the hypothalamus, fibers were found in the lateral and periventricular zones, the ventral hypothalamus close to the ME, and the hypophysis. In the mesencephalon, the periventricular gray zone of the optic *tectum* and the *torus semicircularis*, and in the rhombencephalon fibers were found in the *griseum centrale* and *locus coeruleus* (Berman et al., 2009). This widespread distribution of fibers, covering regions from the anterior telencephalon to the rhombencephalon will be found in mammals and other species. A similar dichotomy between *pmcha*/MCH_A_ and *pmchb*/MCH_B_ is observed in the goldfish (Huesa et al., 2005; Cerdá-Reverter et al., 2006; Matsuda et al., 2006; Tanaka et al., 2009).

These observations of MCH immunoreactivity in Teleostei leading to Euteleostei show a consolidation of the pattern that emerged in ancient Actinopterygii and, in particular, at the time of Lepisosteriform divergence. The NLT group of MCH neurons became the dominant group, and a strong innervation of the ME and the hypophysis developed. On the other hand, the periventricular group started drifting away of the periependymal area and the PVO, and lost contact with the ventricular cavity, but remained in the posteromedial part of the hypothalamus. The duplication of the *pmch* gene also impacted the anatomy of the system. While *pmchb* was retained in all previously described groups of MCH neurons, projections from MCH_B_-synthesizing neurons became concentrated in the hypophysis. On the other hand, *pmcha* became restricted to a small group of neurons in the NLT, but those neurons have widespread fibers in the CNS.

### Teleostei > Euteleostei > Protacanthopterygii

The Euteleosts have been extensively investigated, due to the initial discovery of MCH happening in the chum salmon pituitary. The Euteleosts can be split into two clades: Protacanthopterygii and Neoteleostei, which includes Acanthopterygii (Figure 2). Order Salmoniformes, which includes trout and salmons, is part of the Protacanthopterygii, and will be the focus of this section. The members of the *Oncorhynchus* genus played a historical role in the discovery of MCH since the peptide was first isolated from the hypophysis of the chum salmon (*Oncorhynchus keta*) by Kawauchi et al. (1983).

#### Structural and Evolutionary Aspects

Another major genetic shift occurred after the divergence of the Salmoniformes. In these animals, an additional duplication of the *pmch* genes occurred, conforming to the 4R theory, which states that a fourth whole-genome duplication event occurred in the Salmoniform lineage, between 25 and 100 MYA (Allendorf and Thorgaard, 1984), and this is reflected in gene databases (Figure 2). The nomenclature employed by the automated computational analyses, however, is often confusing and should be interpreted with care. In this review, the two copies of *pmcha* will be called *pmcha1* and *pmcha2*, and the two copies of *pmchb* will be called *pmchb1* and *pmchb2*. Detailed information is available for three species of Salmonids, the rainbow trout (*Oncorhynchus mykiss*), the coho salmon (*Oncorhynchus kisutch*), and the Chinook salmon (*Oncorhynchus tshawytscha*), with minimal variation between species. Both *pmcha1* and *pmcha2* are composed of three exons that encode Pmcha precursors that are 144 aa-long for *pmcha2* (*O. mykiss* – XP_021432849.1 {Chromosome 21}; *O. tshawytsha* – XP_024228562.1 {Chromosome 15}) and 146 or 147 aa-long for *pmcha1* (*O. mykiss –* XP_021419487.1 {Chromosome 15}; *O. tshawytsha* – XP_024232986.1 {Chromosome 17}). At the C-terminus is a mature MCH_A_ that is 21 residues-long due to the insertion of two residues at the N-terminal (Glu^1^ and Ala^2^). There are also two conservative substitutions in the N-terminal stretch before the cysteine ring (Leu^4^ replaces Ile^2^ and Glu^5^ replaces Asp^3^) when compared to the carp MCH_A_. A single substitution differentiates MCH_A__1_ from MCH_A__2_ (Ser^19^ replaces Trp^19^). The *pmchb1* and *pmchb2* genes, on the other hand, are very similar: both are intronless genes that code for 132 aa-long Pmchb (*O. mykiss* – XP_021446538.1 {Chromosome 29}, XP_021458034.1 {Chromosome 5}; *O. tshawytsha* – XP_024252615.1 {Chromosome 33}, XP_024237102.1 {Chromosome 20}). At the C-terminus of those preprohormones is a 17 aa mature MCH_B_, with sequence identical to Otocephala MCH_B_.

Attempts to clone MCH transcripts in salmonids, however, did not reproduce what is observed in the automatic annotation of gene databases. Most attempts to identify *pmch* genes in salmonids resulted in the identification of only *pmchb1* and *pmchb2* transcripts (Masao et al., 1988; Minth et al., 1989; Takayama et al., 1989; Nahon et al., 1991; Baker et al., 1995). Genes identified by library cloning agree well to the independent information available at the online repositories. It is unclear, at this point, if all the salmonid *pmch* genes are expressed. One report in *O. tshawitscha* found similar patterns of expression for both *pmchb1* and *pmchb2* (Masao et al., 1988), while in *O. kisutch* and *O. mykiss* only *pmchb2* was found to be expressed, while *pmchb1* appears to be a silent gene (Nahon et al., 1991; Baker et al., 1995; Suzuki et al., 1995; Suzuki et al., 1997). Further studies are necessary to validate the automatic annotation reports of *pmcha1* and *pmcha2* in salmonids, and to detect if those genes are expressed.

#### Anatomy

The distribution of MCH_B_-ir cells has been reported for *O. mykiss* and *O. keta*. In these animals, the main group of MCH_B_-ir neurons is found in the NLT, encircling the pituitary stalk (Figure 3). A dense network of fibers is directed to the hypophysis, while a few projections are found in the telencephalon, preoptic area, thalamus, and pretectal region. In the hypophysis, fibers predominantly innervated the NH, but could also be found in the AH. Smaller groups of neurons were found behind the pituitary stalk, extending between the basal hypothalamus and the LVR, and medially over the dorsal surface of the LVR, in close contact with the PVO (Figure 3; Naito et al., 1985; Bird et al., 1989; Baker et al., 1995; Suzuki et al., 1995; Baker and Bird, 2002). *In situ* hybridization supports the presence of cell bodies in the NLT and dorsal to the LVR (Baker et al., 1995). This distribution is highly compatible with that described for MCH_B_ in zebrafish and other species of Otocephala.

### Teleostei > Euteleostei > Neoteleostei

The Neoteleostei is a large and complex clade of Euteleosts. Regarding the MCH system, all works have concentrated in a single taxon, Acanthomorpha, and within it, clade Percomorpha of superorder Acanthopterygii. The animals evaluated regarding the MCH system can be divided into two major clades, Carangimorpharia and Percomorpharia. Inside Carangimorpharia, order Pleuronectiformes was the first to diverge, followed then by the closely related orders Beloniformes and Cyprinodontiformes (Figure 2). Inside taxon Percomorpha, all species studies were part of order Perciformes.

#### Evolutionary and Structural Aspects

The idea that the second duplication of MCH occurred specifically in the Salmonid lineage is reinforced by the observation of only two copies of *pmch* in Neoteleosts. Neoteleost *pmcha* encodes Pmcha precursors that range from 146 to 150 aa in length. The mature MCH_A_ contained in these precursors also has a variable length, ranging from 21 aa (winter flounder, *Platichthys americanus*) to 25 aa (barfin flounder, *Verasper moseri*). There is no indication that an MGRP can be originated from Pmcha (Tuziak and Volkoff, 2012; Kang and Kim, 2013; Mizusawa et al., 2015). On the other hand, *pmchb* is an intronless gene, encoding a Pmchb protein that is shorter than Pmcha, ranging from 129 aa (*starry flounder, Platichthys stellatus*) to 136 aa (Nile tilapia, *Oreochromis niloticus*). In all available described and predicted cases, MCH_B_ is 17 aa long and mostly conserved: tilapia MCH_B_ is identical to Otocephala MCH_B_, while other Neoteleosts have a single conservative substitution (Asn^2^ replaces Thr^2^), and the winter flounder has an additional substitution inside the ring sequence (Gly^7^ replaces Val^7^). In most cases, a large 22 or 23 aa-long MGRP precedes mature MCH_B_, potentially cleaved in a single basic *locus* to originate smaller peptides (Gröneveld et al., 1993; Takahashi et al., 2004; Pérez-Sirkin et al., 2012; Tuziak and Volkoff, 2012; Kang and Kim, 2013; Hosomi et al., 2015). It should be noted, however, that Takahashi et al. (2004) found no evidence of MGRP synthesis using mass spectroscopy in *V. moseri* samples.

#### Anatomy

The distribution of MCH has been examined in Pleuronectiformes (Baker and Bird, 2002; Amano et al., 2003; Amiya et al., 2008a, b), Cyprinodontiformes (Batten and Baker, 1988; Batten et al., 1999) and Perciformes (Mancera and Fernández-Llebrez, 1995; Batten et al., 1999; Duarte et al., 2001; Baker and Bird, 2002; Pandolfi et al., 2003; Cánepa et al., 2008). The overall distribution of MCH neurons remains mostly the same, with the main group of cells found in the NLT and a second, smaller group found close to the LVR (Figure 3). The principal group of fibers courses toward the hypophysis, with fibers reaching not only the NH but also the AH junction in the rostral and proximal *pars distalis*. Within the hypophysis, MCH-immunoreactive fibers contact multiple cellular types. A large number of fibers contain stained vesicles in the posterior neurohypophysis, making contact with pituicytes or the basement membrane of capillaries. Discontinuities within the neuro-intermediate basement membrane allow MCH-ir fibers to contact *pars intermedia* endocrine cells, including α-melanocyte-stimulating hormone (α-MSH) and somatolactin (SL) cells. In some instances, MCH fibers are observed in the adrenocorticotropic hormone (ACTH) cell zone and contacting growth hormone (GH) cells. Other cell types do not appear to be contacted by MCH-immunoreactive fibers (Batten and Baker, 1988; Batten et al., 1999). Smaller numbers of fibers are found projecting to other areas, such as the preoptic hypothalamus, thalamus, pretectal region, and telencephalon. In the sailfin molly (*Poecilia latipinna*), fibers are also found in the ventral telencephalon and olfactory bulb (Batten and Baker, 1988). Neurons above the LVR appear to project preferentially to non-hypophyseal targets. At least in Pleuronectiforms, neurons in the NLT are contacted by both gonadotropin-releasing hormone (GnRH)-ir and orexin-ir fibers, but reciprocal connections are only made to orexin neurons (Amiya et al., 2008a, b).

One interesting aspect of the MCH anatomy in Neoteleostei is the description of time-sensitive neurons during development. These neurons appear not to be present in Pleuronectiformes (Amano et al., 2003), but they are found in at least two Perciform species, the gilt-head bream (*Sparus aurata*) and the Cichlid *Cichlastoma dimerus*. In *S. auratus*, cells were found in the periventricular area of the medial hypothalamus from days 4 through 23 after hatching, disappearing after this time frame (Mancera and Fernández-Llebrez, 1995). In *C. dimerus*, the transient neurons were found in the *nucleus periventricularis* posterior, starting at day 6 and disappearing by day 42 after hatching (Pandolfi et al., 2003).

## The Mch System in the Lobe-Finned Fish Lineage

### Osteichthyes > Sarcopterygii > Dipnoi

**FIGURE 4:**
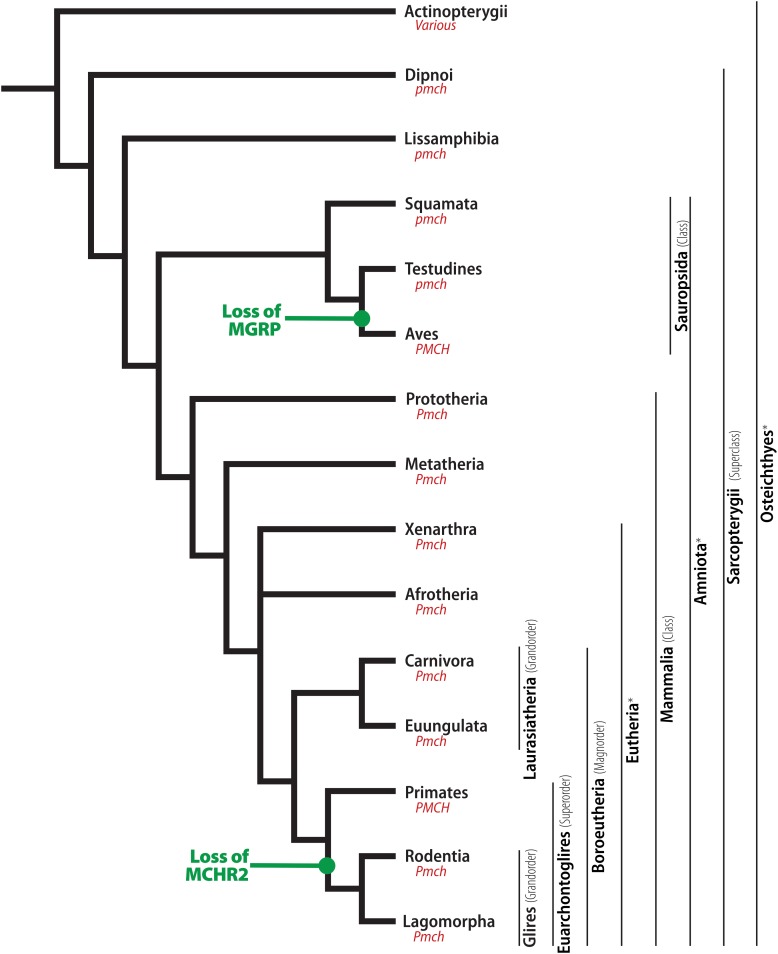
Phylogenetic relationship between Sarcopterygians. Illustrative cladogram showing the main phylogenetic relationship between clades with available information about MCH. Several clades have been omitted for clarity, and the cladogram is not meant to represent the absolute order of relationship between these groups, but rather the most probable organization based on current molecular data and information about the MCH system. Two changes in the MCH system are indicated in the tree: the loss of an MCH gene-related peptide (MGRP) in birds and the loss of the MCH receptor subtype 2 (MCHR2) in Glires. Under each clade name is the standardized nomenclature for the pmch gene that respective clade. ^∗^Unranked clades.

**FIGURE 5:**
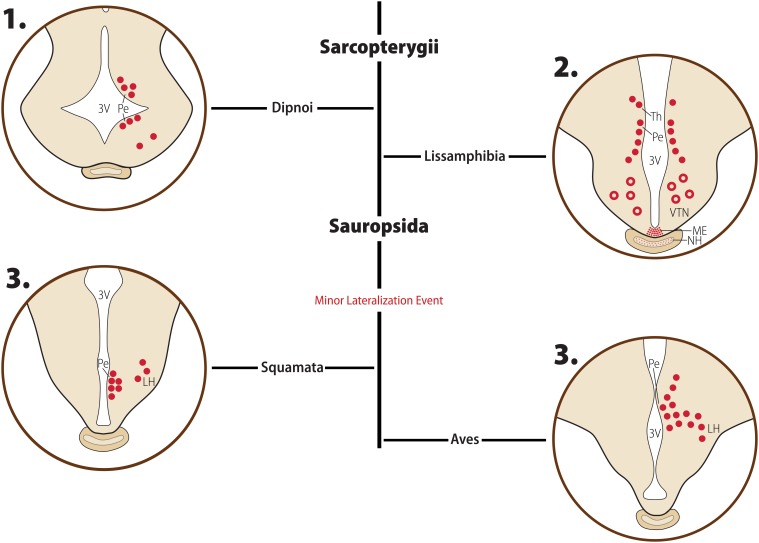
Diagrammatic representation of the main morphological features of the MCH distribution in non-Mammalian Sarcopterygians. Major clades are indicated in bold font, while clade-wide events are represented in red. The diagrams for each clade are not meant to represent a single species or a single hypothalamic level, but rather a visual summary of what has been described for animals belonging to that clade. Full red circles represent MCH neurons, open red circles represent inconsistent information in the literature about MCH-ir neurons in the area. Fiber plexuses are represented by red dots, and inconsistent data about innervation is represented by red stars. **1.** Lungfish (Dipnoi) have a distribution of cells that closely resembles that of Elasmobranchs and early Actinopterygians, with neurons concentrated around the ventricle (Pe), and only scattered cells found in lateral areas. **2.** In Lissamphibians, neurons are still concentrated in the dorsomedial hypothalamus, but these cells are not as close to the ventricle as in other species, and some of the extend dorsally toward the ventral thalamus (Th). Fibers are observed in the Lissamphibian median eminence (ME). There is no consensus about the presence of MCH neurons in the ventral tuberal nucleus (VTN), represented by red open circles, or about the innervation of the neurohypophysis (NH), represented by red stars. **3.** In Sauropsids, a lateral migration event occurred, with some neurons found forming an arc shaped in the lateral hypothalamus (LH). These neurons do not surpass the number of periventricular neurons, however, making this a minor lateral migration event. Based on data from [opetwcite]B137,B35[clotwcite]Vallarino et al. (1998); Cardot et al. (1994), [opetwcite]B36,B58[clotwcite]Cardot et al. (1999); Francis and Baker (1995), and Lázár et al. (2002).

As mentioned before, Actinopterygii and Sarcopterygii diverged at approximately 435 MYA (Kumar S. et al., 2017). The Sarcopterygii clade includes the tetrapod lineage, in addition to the clade containing their closest extant relatives, the lungfish, grouped in Subclass Dipnoi (Figure 4). Limited information is available about MCH in lungfish, as a single work has examined the distribution of MCH-ir elements in the West African lungfish (*Protopterus annectens*) (Vallarino et al., 1998). In this animal, diencephalic MCH neurons are found in two groups: a main group, located in the periventricular tuberal hypothalamus, and a second group described in the peripheral layers of the ventral hypothalamus (Figure 5). According to Croizier et al. (2013), this peripheral group of neurons corresponds to a migrated sheet of cells in contact with the dorsal periventricular hypothalamus. Another two groups have been found in lungfish: in the *subpallium* and the *pars intermedia* of the hypophysis. It is unclear, at this moment, if those extra-diencephalic groups represent actual *loci* of MCH neurons, with further studies necessary to ascertain their specificity. Regarding the fiber distribution, immunoreactive projections are found throughout the telencephalon, including the anterior olfactory nucleus, medial *subpallium*, and medial *pallium*. The preoptic, suprachiasmatic and caudal hypothalamus contain large numbers of fibers, while the thalamus receives a moderate-to-low number of fibers. The mesencephalon and rhombencephalon contain average numbers of fibers, except for the mesencephalic *tectum*, which received a large input, agreeing to what has been described for Actinopterygii. No fibers were observed in the ME or the hypophysis (Vallarino et al., 1998).

### Sarcopterygii > Tetrapoda > Lissamphibia

Tetrapods diverged from lungfish at approximately 413 MYA, and subclass Lissamphibia was the first group to diverge, including all extant amphibians, at approximately 350 MYA (Figure 4; Kumar S. et al., 2017). Amphibians, also classified as anamniotes, are semiaquatic, laying their eggs in the water. Subclass Lissamphibia is composed of three major groups: Gymnophiona (caecilians), Anura (frogs), and Caudata (salamanders). In terms of genetic composition, there are no major changes in Lissamphibians when compared to non-Teleost groups. A single *pmch* gene is found, encoding a Pmch precursor that ranges from 167 to 180 aa in length (*X. tropicalis* – XP_002936876.1, *N. parkeri* – XP_018415182.1). At the C-terminus of this Pmch is a mature MCH identical to mammalian MCH, which can be cleaved from a dibasic Arg-Arg pair. MGRPs can potentially be cleaved from a dibasic pair upstream of mature MCH, but they vary in length and sequence.

The distribution of MCH neurons in Lissamphibians follows a particular pattern that is not observed in other clades (Andersen et al., 1986; Francis and Baker, 1995; Lázár et al., 2002; Croizier et al., 2013). The major cluster of MCH neurons occurs in the dorsal periventricular nucleus, arranged as a subependymal sheet of cells. Although these neurons are found close to the PVO, they do not invade its limits. A second group of cells has been described in the ventral tuberal nucleus, at least in the common frog (*Rana temporaria*) (Figure 5). The lateral hypothalamus (LH) appears not to contain MCH neurons, at least in Anurans. Andersen et al. (1986) describe MCH-ir neurons in the ventral thalamic area of the marsh frog (*Rana ridibunda*), and Lázár et al. (2002) found MCH-IR in the posterior *tuberculum*, which Croizier et al. (2013) insightfully pointed out as harboring dopaminergic neurons. It is possible that these two groups represent the basis of what later became the zona incerta (ZI) and incerto-hypothalamic area (IHy) in mammals (Bittencourt et al., 1992; Sita et al., 2007). There appears to be substantial plasticity in the MCH system of Anurans, with an enlargement of certain subsets of neurons upon the transition from tadpole to adult and the appearance of neurons in the preoptic hypothalamus linked to the reproductive period (Francis and Baker, 1995). Areas that receive MCH input in Lissamphibia include the olfactory lobe, the habenular nucleus, the optic *tectum*, the ME, and the spinal cord. In addition to those areas, Andersen et al. (1986) found a dense plexus of fibers in the NH.

### Tetrapoda > Amniota > Sauropsida

Amniota is a group of vertebrates who have developed adaptations to lay their eggs in a terrestrial environment. The amnion membrane that gives name to the clade is a structure in the egg that forms a cavity filled with fluids around the embryo, providing the necessary hydration during development. This adaptation occurred at approximately 350 MYA and was a key development in the transition from an aquatic to a fully terrestrial life for vertebrates (Kumar S. et al., 2017). At approximately 320 MYA, the Amniota lineage split into Diapsida, who then later originated the Mammalia clade, and Sauropsida, which contains all extant reptiles and birds. Within the Sauropsida clade, order Squamata was the first to split, at approximately 280 MYA, and contains the extant lizards and snakes. The next split within Sauropsida was between Testudines and Archosauria, at approximately 250 MYA. Testudines contains the extant turtles, while Archosauria contains orders Crocodilia and Aves, which split at around 240 MYA (Figure 4; Kumar S. et al., 2017).

The genetic makeup of the MCH system remains strongly conserved in Sauropsida. Among Squamata, most animals from this clade have a single *pmch* formed by three exons that codes for a PMCH that is 164 aa-long in Serpentes (*N. scutatus* – XP_026550810.1, *P. bivittatus* – XP_007420018.1), and between 166 and 174 aa in other clades (*G. japonicus* – XP_015278938.1, *P. muralis* – XP_028601891.1). At the C-terminus of PMCH is a 19 aa-long mature MCH, which is identical to mammals in the common wall lizard (*Podarcis muralis*) and Schlegel’s Japanese gecko (*Gekko japonicus*), or have a single substitution in residue 19 when compared to both humans and Elasmobranchs (Ala^19^ replaces Ile^19^ or Val^19^). An additional substitution is observed in family Elapidae of serpents, where Leu^4^ replaces Met^4^. In Testudines and Crocodilia, PMCH is similar to Squamata PMCH, 167 aa in length and with an MCH sequence that is identical to mammalian MCH (*P. sinensis* – XP_006138097.1, *C. porosus* – XP_019400295.1, *A. sinensis* – XP_006019570.1). In most species within Aves, the mature MCH structure is similar to the other Sauropsids, with occasional changes occurring in individual lineages, especially in positions 4 (Ile^4^, Thr^4^ or Lys^4^ replacing Met^4^) and 19 (Ile^19^ or Ala^19^ replacing Val^19^). It is likely, therefore, that positions 4 and 19 represent “hotspots” that were frequently interchanges during evolution. In non-Avian Sauropsids, an MGRP that is 13 aa in length can be produced from a dibasic site, but in Aves, one of the Arg residues of this cleavage *locus* was replaced by a Glu residue. It is still possible that an MGRP may be produced from a single basic residue by different prohormone convertases, but this seems unlikely (Cardot et al., 1999).

Immunoreactivity to MCH has been described in a few Squamata and Testudines species: the common wall lizard (*P. muralis*), the grass and viperine snakes (*Natrix natrix* and *Natrix maura*), and the water turtle (*Chrysemis scripta elegans*), by Cardot et al. (1994). In Aves, species examined include chicken (*Gallus gallus domesticus*), guinea hens (*Numida meleagris*), quails (*Coturnix coturnix japonica*), gosling (*Anser domesticus*), ducks (*Cairina moschata*), and coots (*Fulica atra*) (Cardot et al., 1999). Cells immunoreactive to MCH were found in two major groups: at the dorsomedial periventricular nucleus, ventrolateral to the PVO, and in the LH. In some cases, a few cells were observed within the PVO. In the LH, cells were described to form an arc shape (Figure 5).

Regarding the distribution of fibers, non-avian Sauropsids are very similar to what is observed in mammals. Fibers are found in olfactory areas, such as the olfactory bulb, the olfactory *tuberculum*, and the piriform cortex; the septum, the diagonal band of Broca, the *paleostriatum*, the amygdala, parts of the cortex, the preoptic hypothalamus, the lateral zone of the hypothalamus, the pretectal area, the optic lobes and in several areas of the brainstem and spinal cord (Cardot et al., 1994). Immunoreactivity was also observed in the same areas when an anti-NEI antiserum was used, confirming the synthesis of an MGRP in non-avian Sauropsids. In Aves, the same basic plan was observed, but a few key differences are noted: projections to the olfactory system are more restricted in birds, the hippocampus receives less MCH fibers, the thalamus receives less dense projections, and although dense, projections to the brainstem are more constrained to specific areas, as opposed to the more diffuse projections observed in other Sauropsids. No staining was observed when an anti-NEI antibody was used (Cardot et al., 1999).

Summarizing these observations, non-avian Sauropsids developed a very similar pattern of projections to mammals, an example of convergent evolution facilitated by a shared common plan first observed in Lissamphibia. In the Aves lineage, however, projections were trimmed in some areas, while developed in others, likely to better work for the different needs of birds in their environment. Another important event was the loss of an MGRP in the Aves lineage, which combined to the great variability in the sequence of those peptides in other *phyla*, raises questions about the extent of functions performed by those peptides. We cannot discard the possibility, however, that the loss of an MGRP in the base of Aves has facilitated the involution of that system in areas where its interaction with MCH was important.

### Tetrapoda > Amniota > Mammalia

Mammals originated from the sister clade of Sauropsida, Synapsida. Mammals are characterized by the acquisition of several morphological traits, including mammary glands, three bones in the inner ear, and hair. Another important development was the acquisition of a placenta, after 160 MYA, which separates the Eutherians from Prototherians (e.g., Platypus and Echidna) and Metatherians (e.g., modern marsupials) (Figure 4; Kumar S. et al., 2017). Among Eutherians, geographically distinct clades developed in between 105 and 100 MYA, including Xenarthra (e.g., anteaters and armadillos), Afrotheria (e.g., moles, shrews, tenrecs, manatees, and elephants), and Boroeutheria (e.g., rodents, primates, carnivores, ungulates), which likely split almost simultaneously (Figure 4; Nishihara et al., 2009). To us, the exact relationship between these groups is of little importance, as almost no information is available for Xenarthra and Afrotheria regarding the MCH system.

Regarding the *Pmch* gene, mammals have a very homogenous composition. The *Pmch* gene is formed by three exons and two introns, which contribute similarly to PMCH formation as in other vertebrates. Mature MCH is 19 aa-long, processed from a dibasic (Arg-Arg) pair, ending with a Val^19^ in most mammals, but a few species have an Ile^19^ (similar to Elasmobranchs), such as the platypus (*Ornithorhynchus anatinus* – XP_001508069.2), the pangolin (*Manis javanica –* XP_017536842.1), and a few primate species, such as the northern white-cheeked gibbon (*Nomaseus leucogenys –* XP_030677139.1) and the white-tufted-ear marmoset (*Callithrix jacchus –* XP_002752955.1). The only exception to that rule are some species of bats, where Ile^5^ replaced Leu^5^ (*E. fuscus* – XP_008142972.1, *M. brandtii* – XP_005880777.1). All substitution observed are conservative and happened outside the loop between Cys residues. Two MGRPs appear to be encoded in mammalian *Pmch*. Mature NEI is 13 aa-long and precedes MCH in the PMCH sequence. The sequence of NEI has been mostly conserved in mammals, with some variation observed in the first three residues, in particular in position 2. These changes are conservative in all cases, except for the rhinoceros (*Ceratotherium simun simun –* XP_014635845.1), where Glu^1^ was replaced by a Gly^1^. While NEI is produced from a dibasic Arg-Arg pair in Prototheria and Metatheria, Eutheria has a single substitution on the first Arg of the pair, allowing NEI to be cleaved from a Lys-Arg pair instead. Finally, NGE may be cleaved from a single basic residue, but evidence of its actual synthesis is lacking (for a review, see Bittencourt and Diniz, 2018).

The distribution of MCH-ir perikarya among mammals is familiar, but distinctive. Here, we observe a significant shift of MCH cells, which now are more numerously found in the LH, rather than the periventricular zone. This lateral migration is similar to what happened during the Actinopterygii differentiation and represents a second, independent shift in the position of MCH cells. Similar shifts occurred with less intensity in other clades, such as Dipnoi and Sauropsida, but the number of cells in lateral areas never surpassed the density of cells in the periventricular area in those groups. While the later migration of MCH cells in Actinopterygii occurred in parallel to an increase of MCH innervation of the hypophysis, the lateral migration observed in Mammals occurred concomitant to an expansion of MCH innervation throughout the CNS. These movements are strongly linked to hodological characteristics of the lateral areas: while the NLT has an intimate relationship with the *infundibulum*, the lateral hypothalamic area (LHA) of mammals acts as the bed nucleus of the medial forebrain bundle (*mfb*), a massive fiber bundle that connects the basal telencephalon to the hindbrain through ascending and descending fibers (Nieuwenhuys et al., 1982). The presence of MCH neurons in the LHA serves a double purpose: it allows these neurons to receive a massive amount of information, at the same time granting access to distant areas of the CNS.

The Mammalian LHA is a large structure, with a very intricate pattern of parcelation based on its connections and neurochemistry (Swanson et al., 2005; Hahn, 2010). Melanin-concentrating hormone-ir neurons are found in the LHA of all studied Mammals, but its relative position within this structure varies. This is likely related to the particular area of the *mfb* that needs to be accessed by MCH neurons, since the *mfb* has a well-defined topographical distribution within the LHA. The LHA group of cells also varies substantially in its rostrocaudal and mediolateral extent among Mammalian clades. Usually, a second diencephalic group is present, in the form of a dorsomedial cluster of cells, which varies among species in terms of its proximity to the ventricle and rostrocaudal position and extent. A ventral thalamic group is often observed, corresponding to the *zona incerta* (ZI). Other groups besides these three will be highlighted for the appropriate species in the next sections.

### Boreoeutheria > Laurasiatheria

Approximately 10 million years after the divergence of Boroeutheria, this clade split again into two groups: Laurasiatheria and Euarchontoglires. Laurasiatheria contains a diverse group of animals, including ungulates, bats, whales, and carnivores. There are only three species of Laurasiatherians with a described distribution of MCH neurons: the domestic cat (*Felis catus*), a Carnivore, and the Euungulates domestic pig (*Sus scrofa domesticus*) and sheep (*Ovis aries*) (Figure 4).

**FIGURE 6:**
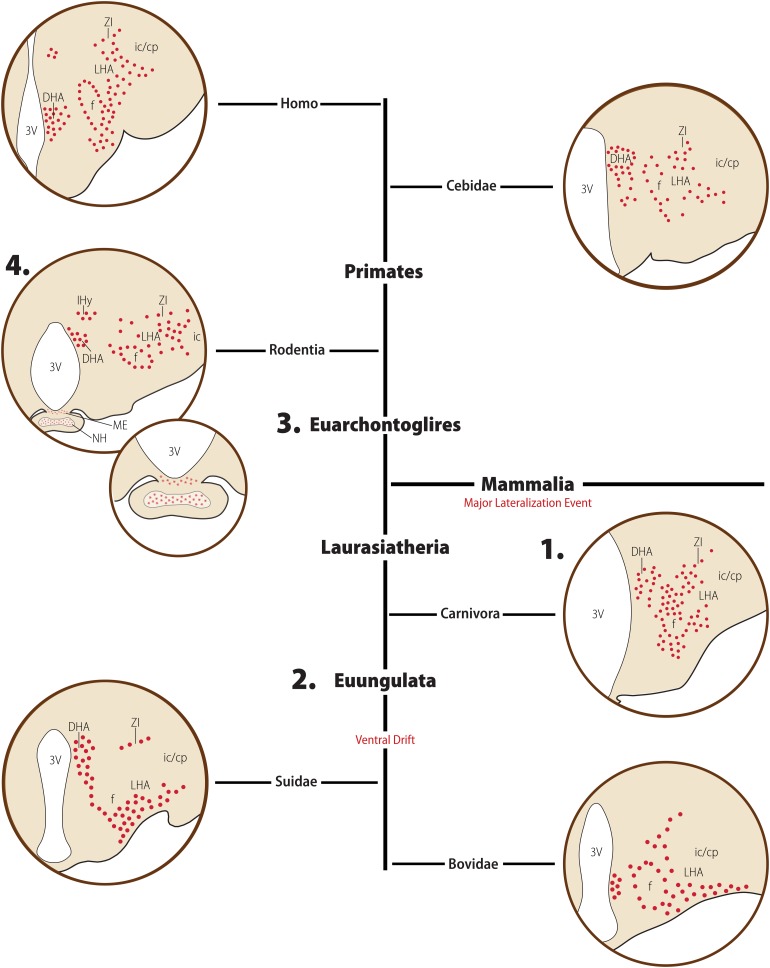
Diagrammatic representation of the main morphological features of the MCH distribution in Mammals. Major clades are indicated in bold font, while clade-wide events are represented in red. The diagrams for each clade are not meant to represent a single species or a single hypothalamic level, but rather a visual summary of what has been described for animals belonging to that clade. Red circles indicate MCH neurons, red dots indicate neurosecretory fibers, and red stars indicate species-specific presence of fibers. The rat was chosen to represent rodents, to avoid the complexity that representing intra-Muroid variations would bring. In the Laurasiatheria branch, numerous MCH neurons are found in the ventrolateral hypothalamus, representing a ventral shift in this group when compared to Euarchontoglires. **1.** In Carnivora, there is a substantial group of labeled neurons in the medial hypothalamus, including the dorsomedial hypothalamic area (DHA), while the lateral part of the lateral hypothalamic area (LHA) is less densely populated. A few neurons are observed in the neighboring zona incerta (ZI). **2.** A very dense distribution of neurons in the ventral hypothalamus is observed in Euungulates. **3.** In Euarchontoglires, the dorsolateral LHA becomes the main locus of MCH neurons, with fewer neurons found in the medial hypothalamus and ZI. In all Mammals, there is a strong association of MCH neurons and the fornix and a DHA group of neurons, possible representing the ancestral group of periependymal neurons seen in Petromyzontids. **4.** In rodents, an additional group of neurons is observed in the incerto-hypothalamic area (IHy), and MCH-ir fibers are observed in the median eminence (ME) and neurohypophysis (NH) of the rat, but not in the closely related mouse. Based on data from [opetwcite]B134,B132[clotwcite]Torterolo et al. (2006); Tillet et al. (1996), [opetwcite]B42,B27[clotwcite]Chometton et al. (2014); Bittencourt et al. (1992), Bittencourt et al. (1998) and Krolewski et al. (2010).

In Euungulates, MCH neurons have an extensive distribution in the rostrocaudal axis (Tillet et al., 1996; Chaillou et al., 2003; Chometton et al., 2014). This large extent results from the combination of the lateral group starting in the anterior hypothalamus, and the dorsomedial group prolonging into the anterior level of the ventral tegmental nucleus. The main group of MCH neurons in the LHA is found in its ventral part, between the ventral margin of the internal capsule and cerebral peduncle (ic/cp), the fornix (f), and the optic tract (Figure 6). Fewer MCH neurons are observed dorsal to the fornix, in the lateral part of the LHA. In the medial zone, a few immunoreactive cells are found in the internuclear space between the dorsomedial and ventromedial hypothalamic nuclei, while the periventricular zone is mostly devoid of labeling. The dorsomedial periventricular group of MCH cells is first observed within the posterior hypothalamic area (PHA). The most significant difference between pigs and sheep concerns the subthalamic area, as no cells were reported in the sheep ZI, while both the rostromedial and ventral ZI were reported to contain immunoreactive cells in pigs (Tillet et al., 1996; Chaillou et al., 2003; Chometton et al., 2014).

The distribution of fibers in Euungulates is extensive, and the primary projection pathways resemble those observed in Lissamphibians and Sauropsids. The anterior pathway conducts fibers to ventral telencephalic and subtelencephalic structures, including the cortical fields ventral to the rhinal sulcus, the dorsal *subiculum*, the *taenia tecta*, the amygdala, and the medial *septum*. Only a light innervation is observed in the hippocampus proper, and the dentate gyrus is mostly devoid of fibers. A dorsal pathway allows a sparse innervation of the midline thalamic nucleus, the habenular nuclei, and the *subthalamus*. A dense descending pathway takes fibers to most of the midbrain and hindbrain, including the optic *tectum*, the *substantia nigra*, the reticular formation and the periventricular gray matter, up to the dorsal horn of the spinal cord. Local dense hypothalamic projections are also observed, mostly restricted to the lateral zone in sheep, but more widespread in pigs. Some fibers are observed in the external layer of the ME (Tillet et al., 1996; Chaillou et al., 2003; Chometton et al., 2014).

The domestic cat, a Carnivore, shows several distinctive characteristics in its distribution of MCH-ir perikarya when compared to Euungulates. The large rostrocaudal extent is not observed, with neurons restricted to the tuberal and mammillary levels of the diencephalon. At tuberal levels, the largest group of neurons is found in the LHA, and, within it, in the perifornical nucleus. These neurons are mostly found in the medial LHA, directly dorsal and ventral to the fornix, making the distribution of MCH neurons in the cat substantially more dorsal than what is observed in Euungulates. Some neurons are also observed in the ventral ZI. In the medial hypothalamus, neurons are found in the dorsal hypothalamic area (DHA), dorsal to the dorsomedial hypothalamic nucleus, and these neurons appear to be contiguous with neurons in the PHA (Figure 6). Immunoreactive neurons are not found at the level of the mammillary bodies (Torterolo et al., 2006; Badami et al., 2010).

While a pattern is easily observed for Euungulates, it is harder to discern an overarching pattern for Laurasiatherians. The two main group of cells observed in Lissamphibians are likely represented here, the LHA cells corresponding to the ventrolateral group, while the DHA/PHA group corresponds to the dorsal infundibular nucleus surrounding the 3V. In cats, however, the LHA cells migrated dorsally, occupying a dorsomedial position within the LHA. It is hard to determine, however, how representative of the Carnivores as a whole the distribution of cats is, as it is the only species of carnivore mapped to this point. The difference between Carnivora and Euungulata could correspond to the difference between feeding habits between these two clades, but more information is necessary to confirm this.

### Boreoeutheria > Euarchontoglires > Rodentia

**FIGURE 7:**
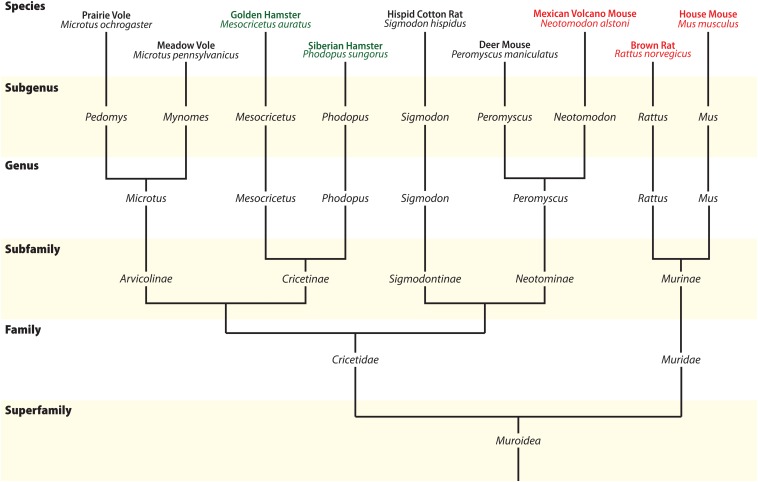
Phylogenetic relationship between Muroids. Illustrative tree showing the phylogenetic relationship between members of Superfamily Muroidea, with several groups omitted for clarity. Commonly used animal models are indicated at the **top** of the tree, and species with available morphological data for MCH are indicated in green and red. Molecular data to build the tree comes from Fabre et al. (2012) and Platt et al. (2015). Reproduced with permission from Diniz et al. (2019).

At approximately 90 MYA, the Euarchontoglires clade split into two major groups: Glires, containing rabbits, hares, and Rodents; and Primates, which includes lemurs, monkeys, and humans. Glires then split into two major orders: Lagomorpha and Rodentia, at approximately 82 MYA (Kumar S. et al., 2017). The Glires clade is marked by the loss of one of the MCH receptor paralogs, making MCHR1 the only receptor found in those animals (Figure 4; Tan et al., 2002). No information about the MCH system is available for Lagomorphs, while Rodents have been amply investigated due to their popularity as animal models. Rodentia is a complex order, with several molecular techniques employed to try to define the relationships between members of this order (Figure 7). Although order Rodentia is composed of several groups, only Muroidea has been investigated in terms of MCH distribution, leaving animals like beavers, squirrels, guinea pigs, and jerboas still to be studied. The two largest groups of Muroids are the Murids and the Cricetids, with these two groups splitting at approximately 33 MYA. The Muroids include the traditional laboratory models, the brown rat (*Rattus norvegicus*) and the house mouse (*Mus musculus*), which split at approximately 21 MYA (Kumar S. et al., 2017). Both rats and mice have been used to describe the distribution of MCH-ir elements (Bittencourt et al., 1992; Elias et al., 2001; Diniz et al., 2019). The Cricetids include a diverse group of species, including voles (Arvicolinae), hamsters (Cricetinae), and deer mice (Neotominae) (Figure 7). The MCH system has been described in two hamsters, the golden hamster (*Mesocricetus auratus*) and the Siberian hamster (*Phodopus sungorus*) (Khorooshi and Klingenspor, 2005; Vidal et al., 2005), in addition to the Neotomine Mexican volcano mouse (*Neotomodon alstoni*) (Diniz et al., 2019).

**FIGURE 8:**
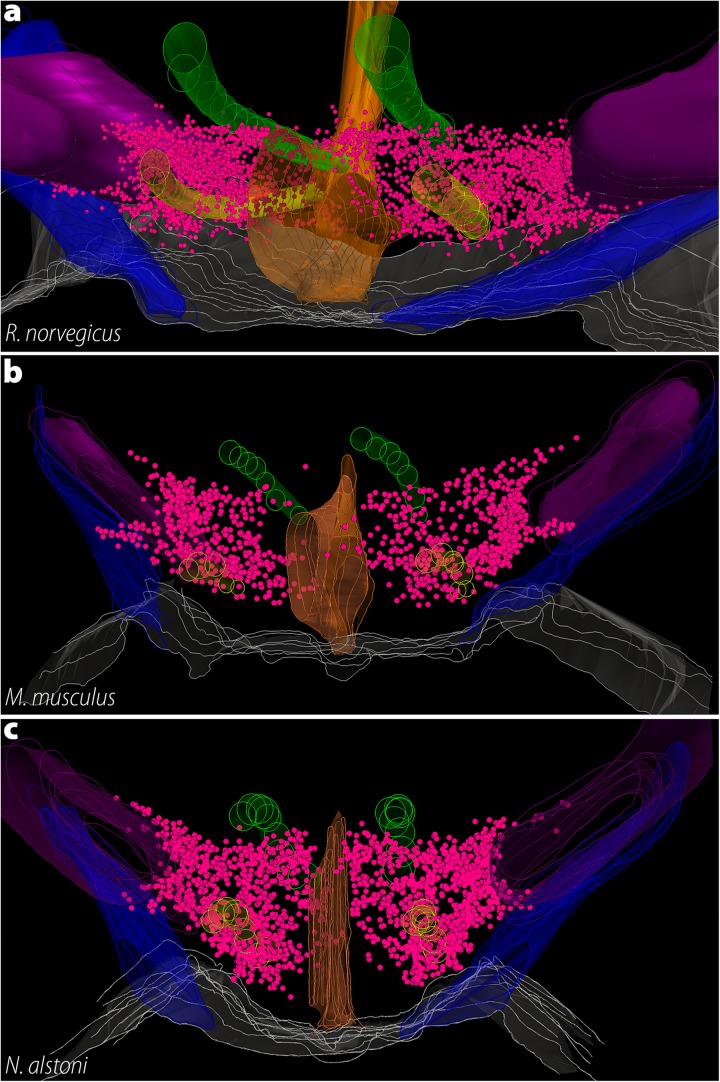
The three-dimensional distribution of MCH neurons in Muroids. Hypothalamic reconstructions from *R. norvegicus* **(a)**, *M. musculus* **(b)**, and *N. alstoni* **(c)**. Each neuron is indicated by a magenta dot. Notable differences are observed when the three species are compared. The distribution of MCH neurons occupies virtually the whole medio-lateral extent of the tuberal hypothalamus, with neurons ranging from the ventricle border to the internal margin of the internal capsule. Only a small number of neurons is found ventral to the fornix. In contrast, the mouse periventricular nucleus and the medial part of the lateral hypothalamic area (LHA) are mostly devoid of neurons, with the anterior hypothalamic area and the dorsolateral LHA representing the largest groups. In *N. alstoni*, an intermediate profile is observed, with moderate numbers of neurons observed in the medial LHA and the periventricular area. The Mexican volcano mouse, however, distinguishes itself for a substantial presence of MCH neurons ventral to the fornix, in the perifornical area. Animated versions of these reconstructions can be found in Diniz et al. (2019). Structures: 3V, third ventricle (orange); f, fornix (yellow); ic, internal capsule (purple); mt, mammillothalamic tract (green); opt, optic tract (blue). Reproduced with permission from Diniz et al. (2019).

As mentioned before, the presence of MCH in the LHA is shared among all Mammals, and Rodents are no exception. In all cases, the largest cluster of MCH neurons is found in the dorsolateral tuberal LHA, lateral to the medial part of the internal capsule and cerebral peduncle (Figure 6). In *R. norvegicus*, *N. alstoni*, and *P. sungorus*, a large number of MCH neurons is observed within the limits of the perifornical nucleus, while *M. musculus* and *M. auratus* have only scattered cells in this area. The mediolateral extent of MCH cells within the LHA is also variable, with a continuous band of neurons from the pericapsular part to the medial hypothalamus observed in *R. norvegicus*, while in *M. musculus* MCH cells are mostly restricted to the area lateral to the fornix. Dorsal to the LHA is a group of cells in the ventral ZI, observed in all Rodent species. The lateral cells are observed until the tuberomammillary level and disappear before the mammillary nuclei are fully formed. A comparison on the three-dimensional distribution of MCH cells in rats, mice, and volcano mice is available in Figure 8.

The medial zone of the hypothalamus, on the other hand, shows more variability between species. At the dorsalmost part of the medial zone of the hypothalamus rests a small group of MCH neurons intermingled with dopaminergic neurons the A13 group, corresponding to the IHy (Sita et al., 2003, 2007). The existence of a neurochemically defined IHy has been demonstrated in *R. norvegicus* (Sita et al., 2003, 2007) and *M. musculus* (Diniz et al., 2019), and has been suggested in *N. alstoni* and *M. auratus* (Vidal et al., 2005; Diniz et al., 2019). Ventral to the IHy is one of the two dorsomedial groups of MCH cells found in Rodents, the anterior hypothalamic area group (AHA). In *R. norvegicus*, these cells are scarce and separated from the IHy, while numerous neurons are found in the AHA of *M. musculus* and *N. alstoni*, and this group is virtually continuous with the IHy. The second group of dorsomedial MCH cells is found in the PHA, in close association with the 3V. This group is observed in *R. norvegicus*, *N. alstoni*, and *P. sungorus*, but not in *M. musculus* or *M. auratus*. In addition to those groups, rats and mice have been shown to have an additional time-sensitive group of MCH neurons that is detectable only in the medial preoptic area, preoptic periventricular nucleus, and anterior paraventricular hypothalamic area of lactating animals (Knollema et al., 1992; Rondini et al., 2010; Alvisi et al., 2016; Ferreira et al., 2017b; Costa et al., 2019; Diniz et al., 2019). Several extra-diencephalic groups of MCH neurons have been identified exclusively in rats. In the basal forebrain, MCH neurons are found in the olfactory tubercle, and in the brain stem, immunolabeling is found in the laterodorsal tegmental nucleus, only in females, and in the paramedian pontine reticular formation (Bittencourt et al., 1992; Bittencourt and Diniz, 2018). Brainstem MCH neurons have also been observed in the cat (Costa et al., 2018). Regarding the distribution of fibers, almost all areas of the rat CNS receive MCH-ir fibers to some extent, except for some motor nuclei of the hindbrain (Bittencourt et al., 1992).

As the available data reveals, a substantial expansion of the MCH system occurred in Rodents. The LHA is the main group of neurons, as in other Mammals, but this group is the most extensive in Rodents, including the whole pericapsular LHA, and often the medial LHA and the perifornical nucleus. This distribution is more lateral than what is observed in Carnivores, and more dorsal than what is observed in Euungulates, and varies substantially among Rodents. As mentioned before, such variability may be linked to preferential access to some parts of the *mfb*, leading to richer innervations of some areas when compared to others. These differences may be linked to the vastly different habits and behaviors displayed by Mammals. The dorsomedial group of cells is also substantially variable within the rostrocaudal and mediolateral axes. In rats, dorsomedial MCH neurons are very close to the third ventricle, and neuroendocrine areas are densely innervated (Bittencourt et al., 1992; Diniz et al., 2019), while in mice these neurons drifted away from the periventricular zone and neuroendocrine areas appear to receive fewer fibers (Diniz et al., 2019).

Although the contact between MCH and the ventricular lumen first observed in Petromyzontids appears to have been lost in the early Sarcopterygii lineage, at least in Rodents, this contact appears to have been reacquired, resulting in a volume transmission mode of communication for MCH neurons (Noble et al., 2018). Other aspects of the distribution of MCH neurons in Rodents may have counterparts in distant species, including the time-sensitive appearance of MCH neurons linked to reproductive stage (Petromyzontids, Anurans), neurons in the basal forebrain (Dipnoi), neurons in the brainstem (Carnivora), and neurons in a transitional structure between the hypothalamus and the subthalamus, in proximity to dopaminergic neurons (Anurans). The multiple independent acquisitions of the same morphological aspects indicate that, despite the frequent occurrence of losses, it is likely that underlying properties of the peptidergic system facilitate the reacquisition of lost characteristics, despite substantial divergence in terms of habitat and behavior among species.

### Boreoeutheria > Euarchontoglires > Primates

Only two species of Primates have been examined in terms of MCH morphology: the tufted capuchin monkey, of the *Sapajus* genus, and humans (Figure 4). *Sapajus* spp. are new-world monkeys, member of Family Cebidae, which diverged at approximately 43 MYA. Member of genus Homo are believed to have split from their closest relatives, Pan, at approximately 6.7 MYA (Kumar S. et al., 2017). It should be noted that, in the original description of MCH in a new world monkey, the species has been identified as *Cebus apella* (Bittencourt et al., 1998). The taxonomic identification of the monkeys used in that experiment has been revised, as those animals are now more closely identified with members of genus *Sapajus*, and there is some controversy in the precise species definition (for a brief discussion on this subject, see Battagello et al., 2017). In *Sapajus* spp., MCH neurons are found exclusively in the diencephalon. These neurons are found from the caudal levels of the paraventricular nucleus up to the level of the medial mammillary nucleus, a distribution in the rostrocaudal axis that is slightly longer than Rodents, but shorter than Laurasiatherians. In the anterior tuberal hypothalamus, cells were observed dorsal to the fornix and in the dorsal part of the periventricular nucleus, in addition to the lateral ZI, but there is no indication that cells are found in the IHy area. At more caudal levels, the main group of neurons is observed occupying the LHA, but the medial zone is devoid of neurons (Figure 6). At mammillary levels, neurons are found dorsal to the medial mammillary nucleus and ventral to the mesencephalic aqueduct. Fibers were found in the medial mammillary nucleus, the external layer of the ME, and the lateral *globus pallidus*, but a complete mapping of fibers has not been published (Bittencourt et al., 1998).

Several authors investigated the distribution of MCH neurons in humans of male and female individuals, both through *in situ* hybridization for *PMCH* (Elias et al., 1998; Krolewski et al., 2010) or immunohistochemistry for MCH (Thannickal et al., 2007; Aziz et al., 2008). Due to how detailed is the description provided by Krolewski et al. (2010), it will be used as a base to describe the distribution of MCH in *H. sapiens*. Neurons expressing *PMCH* mRNA are first detected in the LHA, at the intermediate levels of the PVH. While Krolewski et al. (2010) identified these anterior neurons as belonging to the LHA, Elias et al. (1998) identified them as part of the rostromedial ZI, possibly corresponding to the Muroid IHy. As the PVH nears its caudal end, the distribution of *PMCH* mRNA-expressing neurons expands to the lateral aspects of the LHA, and two new groups are observed: the dorsomedial hypothalamic nucleus and the DHA (Krolewski et al., 2010). Together, these groups give the impression that MCH neurons surround the whole tuberomammillary extent of the fornix (Figure 6; Elias et al., 1998). A dense cluster of MCH neurons in the DHA forms a ring-shaped structure around a central core of non-labeled cells (Krolewski et al., 2010). This observation is particularly interesting, as Diniz et al. (2019) recently proposed that, in *M. musculus*, MCH neurons form a ring-shaped structure in the LHA surrounding ORX neurons, a neurochemical division called the LHA shell. It is possible that a similar feature appears in humans, with MCH neurons surrounding another population of neurons. Finally, at the level of the mammillary bodies, the DHA continues into the PHA, which assumes a position close to the 3V.

In addition to the distribution of neurons, the number of MCH neurons has also been investigated, particularly in studies evaluating hypothalamic correlates in neurodegenerative diseases. Thannickal et al. (2007) report a loss of MCH neurons throughout the anteroposterior axis that is correlated with Parkinson’s Disease stage, ranging from 12% of loss in Stage I to 74% of loss in Stage V. Aziz et al. (2008) performed a similar experiment in Huntington’s Disease patients, but found no loss of MCH neurons in patients as compared to the controls. Regarding immunoreactive fibers, only Elias et al. (1998) reported fibers immunoreactivity, citing areas such as the cingulate and insular cortex, amygdala, hippocampus, anterior thalamic nucleus, preoptic hypothalamus, and mammillary bodies are recipients of fibers. These innervation fields are compatible with what has been described in other mapping studies for mammals.

Considering the data available, the distribution of MCH neurons in Primates appears to be mostly conserved. Both *S. apella* and *H. sapiens* share the same rostrocaudal extent, and the areas containing the most substantial numbers of neurons are the same. Some crucial questions are still open, however. It is unclear, at this point, if Primates have an IHy similar to Murids, where MCH neurons are found intermingled with tyrosine hydroxylase-synthesizing neurons. The second question is the presence of MCH neurons in the ZI, as Bittencourt et al. (1998) reported cells in the lateral ZI of *Sapajus* spp., but the same has not been reported in humans (Elias et al., 1998; Krolewski et al., 2010). Furthermore, there is no complete mapping of MCH fibers in the human brain and no physical evidence that NEI is synthesized in Primates. The overall pattern observed in Primates appears to be unique to this clade, as the rostrocaudal extent of MCH neurons is longer in Primates than it is in Rodents but shorter than what has been described for Laurasiatherians. It is a common feature between Euarchontoglires, however, the predominance of neurons dorsal to the fornix, and a lateromedial presence of neurons that spans the lateral and medial zones, with only the rat presenting a dense cluster of cells in the periventricular nucleus. There is a high number of labeled neurons around the fornix in several Euarchontoglires species (with the exception of mice and golden hamsters), but it is possible that the neurons observed in humans are actually part of other nuclei in the hypothalamus, compressed against the fornix due to the reduced lateromedial extent of the hypothalamus in the Primate lineage.

## Conclusion

The melanin-concentrating hormone peptidergic system is a versatile neurochemical system, demonstrated to play a varied range of roles within the Rodent CNS. Such functional diversity is only possible thanks to a complex morphological substrate, which has been investigated in a substantial number of species. This morphological substrate, however, is not a universal feature, varying among species and clades, likely reflecting differences in habitat and behavior. This interplay between MCH probably developed from the initial function of MCH: broadcaster of external stimuli. By contacting the ventricular lumen with fine projections while projecting to the lateral hypothalamus and thalamus, MCH neurons were able to coordinate metabolic signals present in the CSF with effector centers of the diencephalon. As vertebrates evolved to display more complex behaviors and a richer relationship with their environment, MCH neurons that could integrate not only endogenous signals, but also external inputs were positively selected, and this is reflected in a drift of MCH cells away from the ventricle. This aligns well with the idea that MCH is a major integrator of internal and external information, ensuring an appropriate response to ensure the organism homeostasis. It would not be possible, however, if all organisms shared identical MCH organizations, given the different challenges imposed by different aquatic/terrestrial environments, food chain positions, and reproductive strategies. New studies of the MCH system in species that have not been investigated yet will help us understand more precisely how these habitat changes are connected to the hypothalamic neurochemical circuits, paving the way to new intervention strategies that may be used with pharmacological purposes.

## Author Contributions

GD and JB contributed with the writing of this article and approved it for publication.

## Conflict of Interest

The authors declare that the research was conducted in the absence of any commercial or financial relationships that could be construed as a potential conflict of interest.

## Abbreviations

3V: third ventricle
A13: dopaminergic group A13
aa: amino acid(s)
ACTH: adrenocorticotropic hormone
AH: adenohypophysis
AHA: anterior hypothalamic area
AROM: antisense RNA overlapping MCH
CNS: central nervous system
CRF: corticotropin-releasing factor
CSF: cerebrospinal fluid
DHA: dorsal hypothalamic area
DHN: dorsomedial hypothalamic nucleus (non-mammalian)
f: fornix
GH: growth hormone
GPR24: G protein-coupled receptor 24
ic/cp: internal capsule/cerebral peduncle
IHy: incerto-hypothalamic area
IR: immunoreactivity
LH: lateral hypothalamus
LHA: lateral hypothalamic area
LVR: lateral ventricular recess
MCH: melanin-concentrating hormone
MCHR1: melanin-concentrating hormone receptor 1
MCHR2: melanin-concentrating hormone receptor 2
ME: median eminence
mfb: medial forebrain bundle
MGOP: MCH gene-overprinted polypeptide
MGRP: MCH gene-related peptide
MYA: million years ago
NEI: neuropeptide glutamic acid-isoleucine
NGE: neuropeptide glycine-glutamic acid
NH: neurohypophysis
NLT: lateral tuberal nucleus (*nucleus lateralis tuberis*)
*Npr-24*: neuropeptide receptor family 24 gene
ORX: orexin
PCR: proximal neurosecretory contact region
Pe: periventricular hypothalamic area
PHA: posterior hypothalamic area
*Pmch/pmch/PMCH*: melanin-concentrating hormone precursor (gene)
Pmch/PMCH: melanin-concentrating hormone precursor (protein)
*PMCHL1*: *PMCH*-Linked 1 gene
*PMCHL2*: *PMCH*-Linked 2 gene
PVO: paraventricular organ
SL: somatolactin
SLC-1: somatostatin-like receptor 1
SSTR: somatostatin receptor(s)
Th: thalamus
VTN: ventral tuberal nucleus
ZI: zona incerta
α -MSH: α -melanocyte-stimulating hormone.
